# Elasto-inertial microfluidics for bacteria separation from whole blood for sepsis diagnostics

**DOI:** 10.1186/s12951-016-0235-4

**Published:** 2017-01-04

**Authors:** Muhammad Asim Faridi, Harisha Ramachandraiah, Indradumna Banerjee, Sahar Ardabili, Sergey Zelenin, Aman Russom

**Affiliations:** Division of Proteomics & Nano-biotechnology, School of Biotechnology, Royal Institute of Technology KTH, SciLifeLab Tomtebodavägen 23, 17165 Solna, Sweden

**Keywords:** Micro particle separation, Elasto-inertial microfluidics, Sepsis, Sample preparation

## Abstract

**Background:**

Bloodstream infections (BSI) remain a major challenge with high mortality rate, with an incidence that is increasing worldwide. Early treatment with appropriate therapy can reduce BSI-related morbidity and mortality. However, despite recent progress in molecular based assays, complex sample preparation steps have become critical roadblock for a greater expansion of molecular assays. Here, we report a size based, label-free, bacteria separation from whole blood using elasto-inertial microfluidics.

**Results:**

In elasto-inertial microfluidics, the viscoelastic flow enables size based migration of blood cells into a non-Newtonian solution, while smaller bacteria remain in the streamline of the blood sample entrance and can be separated. We first optimized the flow conditions using particles, and show continuous separation of 5 μm particles from 2 μm at a yield of 95% for 5 µm particle and 93% for 2 µm particles at respective outlets. Next, bacteria were continuously separated at an efficiency of 76% from undiluted whole blood sample.

**Conclusion:**

We demonstrate separation of bacteria from undiluted while blood using elasto-inertial microfluidics. The label-free, passive bacteria preparation method has a great potential for downstream phenotypic and molecular analysis of bacteria.

**Electronic supplementary material:**

The online version of this article (doi:10.1186/s12951-016-0235-4) contains supplementary material, which is available to authorized users.

## Background

Despite progress in medical science, including the development of effective therapies, infectious diseases continue to cause millions of deaths worldwide, and pathogens in food, animals, water, and plants cause damage and production losses running into billions of dollars. To date, pathogens are typically detected only after they have already caused massive damage. Improved diagnostic methods for infectious pathogens are, therefore, urgently needed. For example, sepsis—an acute inflammatory response of immune-compromised patients to certain pathogens—is the third most common cause of death in Germany [1]. In the case of septic shock, studies have shown that patient mortality will increase by 7.6% for each hour the antibiotic therapy is delayed [2], and, if the initial antibiotic therapy is inappropriate, the survival rate decreases from 52 to 10.3% [3]. Therefore, in infectious diseases and sepsis diagnosis the foremost important step is to find the suitable treatment and identification of the bacteria to prevent evolution of resistant bacteria. Currently, blood-culturing method is the gold standard for identification of microorganism. However, the automated method still requires 24–72 h to get the results [4]. This long turnaround time, especially for the identification of antimicrobial resistance, is driving the development of molecular diagnostics, often based on polymerase chain reaction (PCR) and are used to detect pathogens either from blood culture bottles [5–7] or directly from blood [8–13]. While the total time for diagnosis has been shortened significantly, the implementation of these molecular methods in clinics has been severely hampered by their lack of sensitivity in comparison with, for instance, blood culturing and the need for complex, multi-step sample preparation. Some of the factors affecting the quality in nucleic acid-based methods are PCR inhibitors, abundant interfering human DNA, the risk of carryover when processing several samples, inadequate lyses, and pathogens enclosed within or adhering to human cells. Despite improvements, sample preparation remains the bottleneck for the further development and implementation of molecular diagnostics in clinical settings. Hence, molecular diagnostics would benefit from a rapid, integrated sample-preparation assay method to isolate and enrich bacteria from complex sample matrices such as blood.

Microfluidics has the potential of eliminating the shortcomings associated with complex sample preparation. Microfluidics provide a higher surface to volume ratio, a faster rate of mass and heat transfer, and the ability to handle very small volumes of reagents in microchannels very precisely. Moreover, microfluidics open up the possibility for automated platforms with integrated microfluidic cartridges thereby reducing the risk of contamination [9]. Therefore, a recent interest of microfluidic techniques has been towards the separation of microorganisms from blood. Different approaches to separate pathogens from blood using affinity separation [13–15], size [16, 17] or electrokinetic properties [18] have been demonstrated. These methods typically exploit the difference between cell properties, such as the size, shape, density, deformability, electric/magnetic susceptibility, and hydrodynamic properties. Among these parameters, size is an excellent label-free biomarker for bacteria separation from blood.

Very recent, inertial microfluidics has been described as a high-throughput, simple method for precise manipulation particles based on size [19]. Recently, Wu et al. [20], separated bacteria from diluted red blood cells using ‘‘soft” inertial microfluidics that utilized deflection of larger cells in an asymmetrical sheath flow around a curvature while the smaller cells are kept on or near the original flow streamline. While the yield was about 62%, they obtained an impressive high purity of 99.7%. Similarly, Mach et al. [21] used a straight channel to separate bacteria from red blood sample using massively parallel channels. Here, size-dependent inertial lift forces were used to focus larger red blood cells as a method of cell separation and the authors achieved 80% removal of bacteria from diluted red blood cells after two passes of the single channel system.

While promising, the narrow size difference between microorganisms (typically 1–3 µm) and blood cells (3–15 µm) has shown to be very difficult for bacteria separation using inertial microfluidics. In addition, the fact that bacteria are smaller than blood cells and will end up in the “unfocused” stream, and in essence everywhere in the channel cross-section makes it difficult to achieve proper separation. Very recent, Hou et al. [22] used dean flow fractionation to address this by introducing a sheath flow at the inlet to pinch blood sample and size based migration of the blood cells towards the inner wall while the bacteria are lagging behind and could be extracted at an efficiency of 70% [23]. In this work, we address this by employing elasto-inertial microfluidics instead to differentially migrate larger blood cells away from smaller bacteria in flow through straight channels.

Using elasto-inertial microfluidics, it is possible to migrate particles across streamlines and focus into a single stream in three-dimensional channel depending upon their size [24–30]. A number of investigations have recently focused on optimizing different conditions like concentration of non-Newtonian fluids (elastic forces) and flow rates (inertial forces) [31]. The inertial and elastic forces have been used in combination to separate smaller and larger particles from each other [32] and for separation of blood cell components [33]. Elasto-inertial microfluidics was utilized by Liu et al. [34] to separate bacteria (*E. coli*) from red blood cells without the use of sheath flow. However, the channel dimension used is not applicable for other blood cells as the smallest dimension of the cross-section was 10 μm, which would easily clog for applications using whole blood.

In this paper, using elasto-inertial microfluidics, we separate bacteria from undiluted whole blood by selectively migrating blood cells away from the walls towards the centerline of the channel while bacteria are remained in the streamline they enter and separated. We first investigate the elastic and inertial forces theoretically using simulations and experimentally using different sized particles and viscoelastic solutions. We optimized the flow conditions to continuously separate large particles (5 μm) from small particles (2 μm). Following, we applied the optimal flow conditions to continuously separate bacteria from undiluted whole blood.

## Results and discussion

### Elasto-inertial based particle focusing and separation

**Fig. 1:**
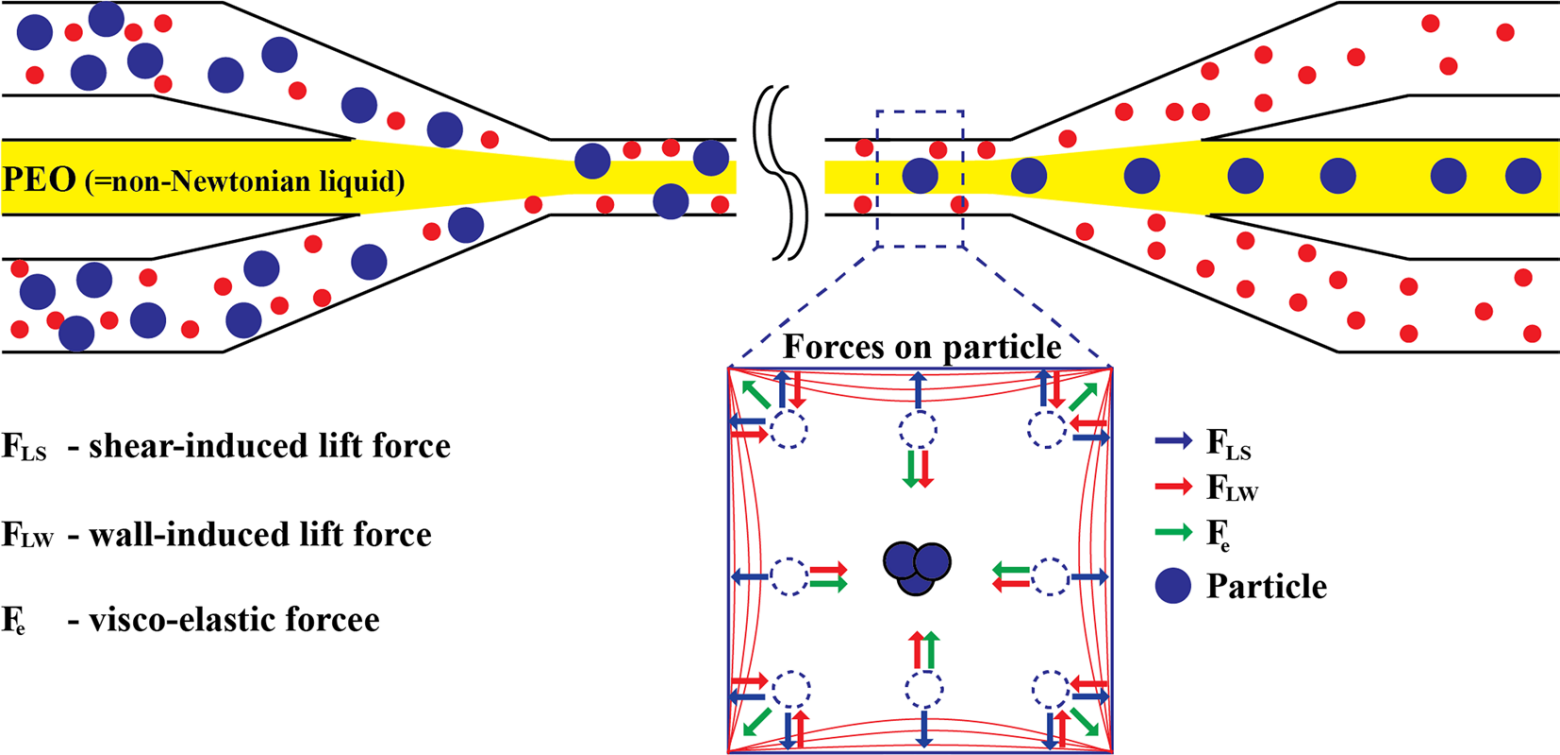
Schematic illustration of elasto-inertial microfluidics based particle migration and separation in flow through straight channel. A mixture of small (*red*) and large (*blue*) particles entered the channel along the sidewalls and non-Newtonian solution is used as sheath at the center. The net result of elastic (F_e_) and inertial forces (shear-induced lift force, F_LS_, and wall-induced lift force, F_LW_) affect the large particles such that they laterally migrate to three dimensional single stream in the middle of channel while the small particles keep flowing along the wall and can be separated

Elasto-inertial microfluidics harnesses a synergetic effect of viscoelastic forces and inertial forces to focus particles based on size (Fig. 1). In pressure-driven viscoelastic flows, the first and second normal stress differences lead to particle migration across the streamlines and occupy multiple equilibrium positions including four at the corners and one at the center in flows through rectangular channels [32]. By moderately increasing the flow rate so that the fluid inertia becomes non-negligible, it is possible to reduce the focusing positions to a single one at the center of the channel (Fig. 1).

To quantitatively evaluate the effect of lift forces (F_L_) and elastic forces (F_e_), it is possible to employ two dimensionless numbers: Reynolds number (Re) and Weissenberg number (Wi). The channel Reynolds number (Re) is defined to describe the magnitude ratio of inertial force to viscous force, while Wi describes the magnitude ratio of elastic force to viscous force (Additional file 1: Figure S1). Particle’s Reynolds number (Rp) is another important dimensionless number that accounts for particle size [Rp = Re (a/D_h_)^2^].

**Fig. 2:**
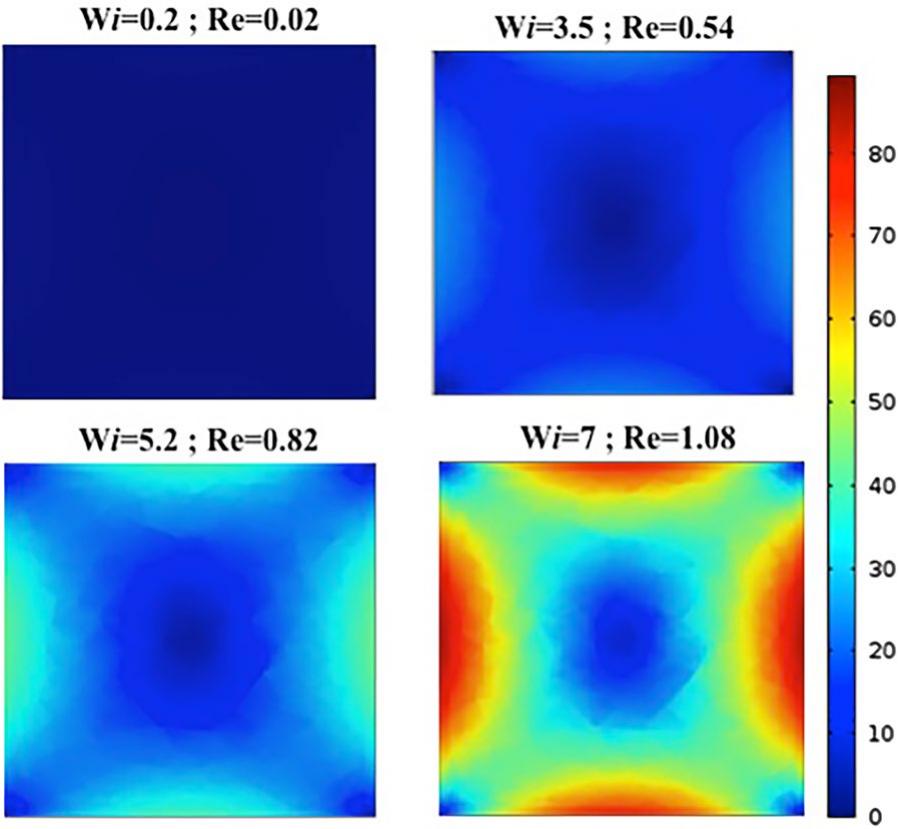
Comsol simulation showing first normal stress difference distribution of non-Newtonian fluid in flow through straight rectangular microchannel. The flow rate is varied from 6 µl/h (Re = 0.017) to 390 µl/h (Re = 1.082) at Weissenberg number (Wi): 0.2, 3.5, 5.2 and 7 respectively

Using COMSOL multiphysics, the First Normal Difference distribution (N1) in non-Newtonian fluid was modelled from very low flow i.e. Re = 0.02 (creeping flow condition) up to Re = 1.08 (elasto-inertial combined regime). As can be seen in Fig. 2, at lower Re and when Wi is close to zero, the fluid is not perturbed notably, due to insufficient elasto and inertial forces and the first normal stress difference is not distributed to induce F_e_ that could affect particle position. Increasing the flow rate increases the shear stress on the non-Newtonian solution, and under stress a non linear viscosity decreasing behavior (shear thinning) will give rise to the regions of higher and lower first normal stresses, distributed such that five equilibrium position for particles are formed [32, 35] most evident at W*i* = 7 in Fig. 2. From the simulation, around W*i* = 3.5 and Re = 0.54 the normal stress distribution differences becomes noticeable. The normal difference distribution effect with respect to flow rate around 5 μl/min is in agreement with previous work of Yang et al. [32].

**Fig. 3:**
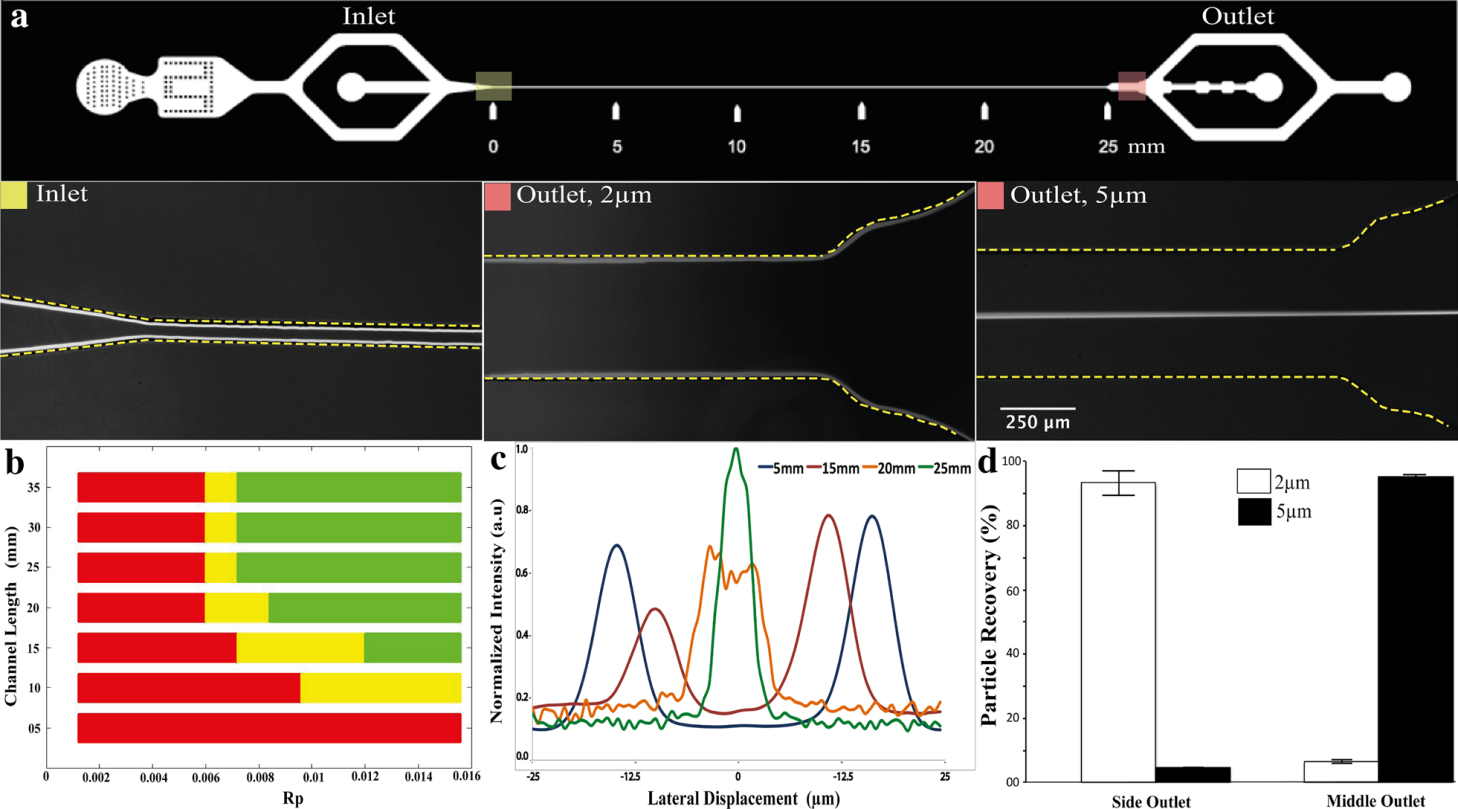
Elasto-inertial particle focusing and separation. **a** Particle focusing and separation in flow through straight microfluidic channel. 2 and 5 µm particle suspension is introduced at inlet (highlighted as *yellow square*). At the outlets (highlighted as *red square*), the 2 µm follow the flow of stream and collected at the side outlets, while the 5 µm particles migrated away from the streamline and focused at the centerline and collected through the center outlet. **b** Focusing profile of 5 µm particles with respect to particle Reynolds number Rp and channel length. The *red*, *yellow* and *green bars* are corresponding to no lateral displacement, semi displacement and complete displacement into the center channel position respectively. **c** Lateral position displacement of 5 µm particles at Rp = 0.008 as the function of length. **d** Separation of 2 and 5 µm particles at different outlets. The yield of the 5 μm particles collected at the middle outlet was 95%, and 93% for the 2 μm particles in the side outlet

Following, we evaluated experimentally the (i) effect of different particle size and channel length; (ii) effect of different concentrations (viscosities) of non-Newtonian fluid (PEO); and (iii) effect of different microchannel dimensions for particle focusing. For the channel geometry, we tested different aspect ratio (approx. 1:1, 1:2 and 1:3) using channel height of 65 μm. For channel aspect ratio 1:2 and above we could not observe particle focusing at the centerline for 10 and 5 µm particles for the channel length tested (=35 mm). The channel aspect ration 1:1 [50 µm × 65 µm (width × height)] resulted in particle focusing at the centerline based on size and was chosen for further investigation (Fig. 3a). Specifically, solution containing the particles (2 and 5 µm) is branched into two streams, one on either side of the entry stream of the non-Newtonian fluid of PEO introduced as a sheath at the center channel inlet. The particles entering the channel at the walls migrate towards the centerline based on size, such that 5 μm particles are fully focused at the center of the channel while the smaller 2 μm particles remain close to the walls and can easily be separated (Fig. 3a). For more systematic analysis of the effect of flow rate on the particle migration, the solution containing 5 µm particles was introduced at a constant flow rate of 30 µl/h while the PEO solution, starting at 300 µl/h was increased at interval of 60 µl/h to cover Re values from 0.25 to 2.1 (corresponding Rp 0.002–0.015 for 5 µm particles). Figure 3b shows the effects of flow are on focusing length for 5 µm particles. A minimum of 15 mm channel length is required to fully migrate 5 µm particles under the given PEO concentration of 500 ppm. As expected, at low flow rate (Rp < 0.006) no focusing is observed presumably due to insufficient elastic forces developed. Furthermore, it is noticeable that a minimum of 25 mm channel length is required to obtain particle focusing when the flow rate is increased (Rp > 0.007). The focusing channel length is further decreased to 15 mm with increased flow rate (Rp > 0.012). As the flow rate is increased further, particle defocusing is observed (see Additional file 2: Figure S2). Particle defocusing is observed at Rp > 0.016, mainly due to shear thinning effect. Furthermore, the effect of non-Newtonian fluid concentration on particle focusing was investigated. While the minimum required channel length was similar (15 mm), as the PEO concentration (viscosity) is increased the focusing is achieved at relatively lower Rp values (see Additional file 3: Figure S3). The 2 μm particles remained unfocused under all the flow rate and PEO concentrations tested, indicating strong *R*p (and hence particle size) dependence for focusing (F_e_ ∝ a^3^ and F_L_ ∝ a^4^).

Next, to mimic bacteria separation, we used the optimized channel dimension, PEO concentration and Re to separate 2 µm from 5 µm particles. The channel dimensions 50 µm × 65 µm (width × height) and PEO concentration of 500 ppm was used to achieve size-based separation of the particles. At the sample flow rate of 30 µl/h and PEO flow rate of 360 µl/h (corresponding to Rp 0.008), the minimum channel length to fully focus 5 µm particles was found around 25 mm (Fig. 3c). Using this channel length and flow conditions, we could successfully separated 5 µm particles from 2 µm particles (Fig. 3d). The yield of the 5 μm particles, calculated as fraction of 5 μm particles recovered through the middle outlet to the total count, was 95%, and the yield was 93% for the 2 μm particles in the side outlet.

### Bacteria separation from whole blood

**Fig. 4:**
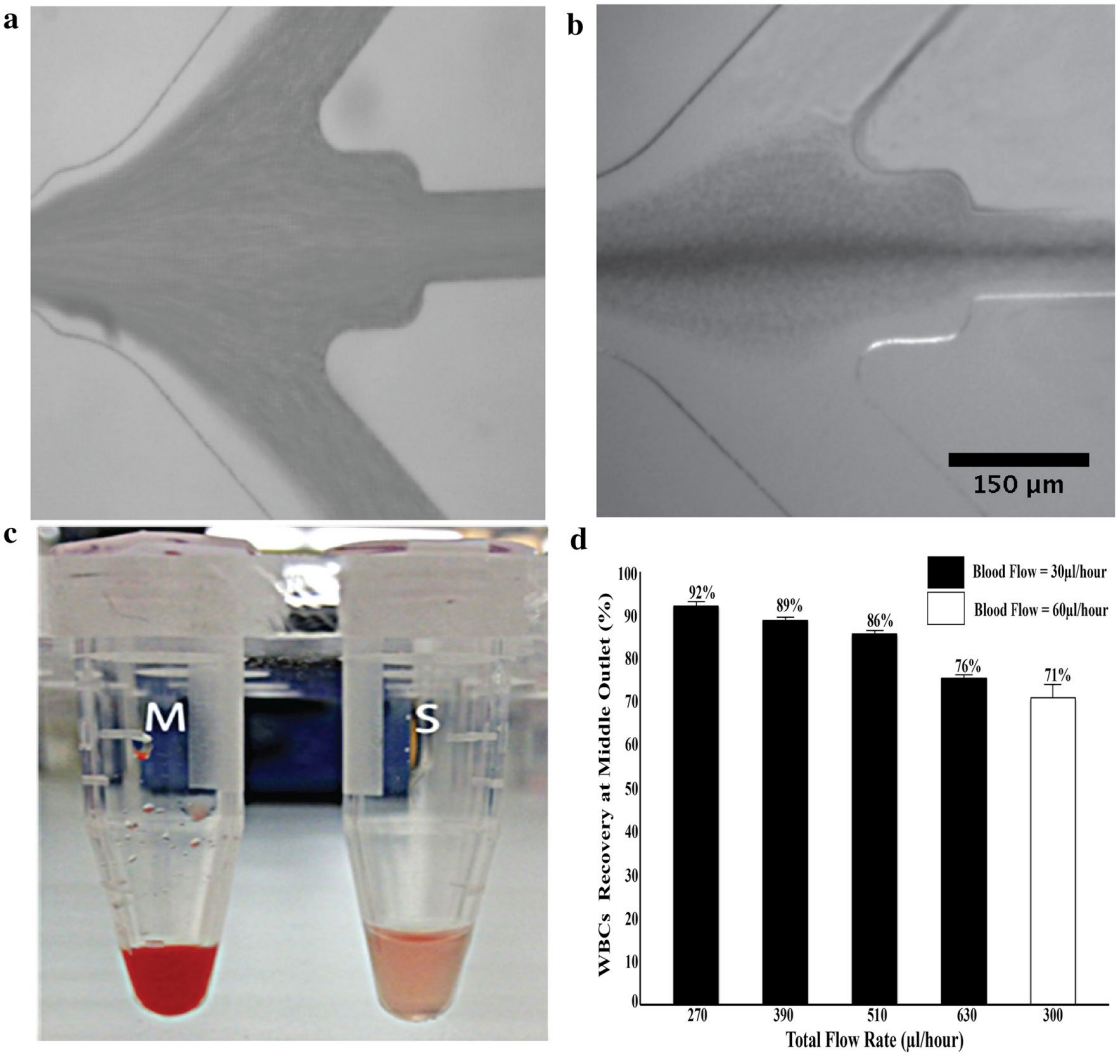
Elasto-inertial focusing of blood cells. Whole blood introduced from side inlet and Newtonian solution (**a**) or non-Newtonian (**b**) is introduced from middle inlet. No lateral migration of blood cell is observed for Newtonian solution, while for the non-Newtonian (PEO) fluid, blood cells are migrated away from the side channel towards the centerline of the channel. **c** The collected sample from middle (*M*) and side (*S*) outlets. **d** Quantification of the blood cells migration at different flow rates. The blood cells migration efficiency towards the middle outlet, analyzed by Coulter counter, was between 76 and 92% depending on flow rate. When the blood flow rate was increased from 30 to 60 µl/h, the efficiency was decreased to 71%

Before bacteria separation from whole blood, we fist investigated the effect of non-Newtonian solution on migration of the blood components. As can be seen in Fig. 4a, when Newtonian fluid (1× PBS) is used, there is no selective migration of blood cell components towards the centerline. However, using PEO we could achieve migration of the blood components, where the blood cells are focused at the centerline and can be extracted (Fig. 4b). As can be seen in Fig. 4c, while the total volumes are similar, the color from the center outlet indicates highly concentrated blood cell components. Furthermore, using Coulter counter we analyzed the WBCs from the two fractions collected over a range of flow rates and obtained a separation efficiency of 92% into the middle outlet (Fig. 4d). The migration of the blood cells is affected by the total flow rate as well as the relative inlet fraction of the whole blood to the PEO sheath buffer. For instance, when the flow rate increases, insufficient migration of the cells results in reduced separation efficiency through the middle outlet. These results clearly show that non-Newtonian fluid can effectively be used to separate blood components, and could find several applications including plasma separation from blood. Here, we have applied this for bacteria separation from whole blood.

**Fig. 5:**
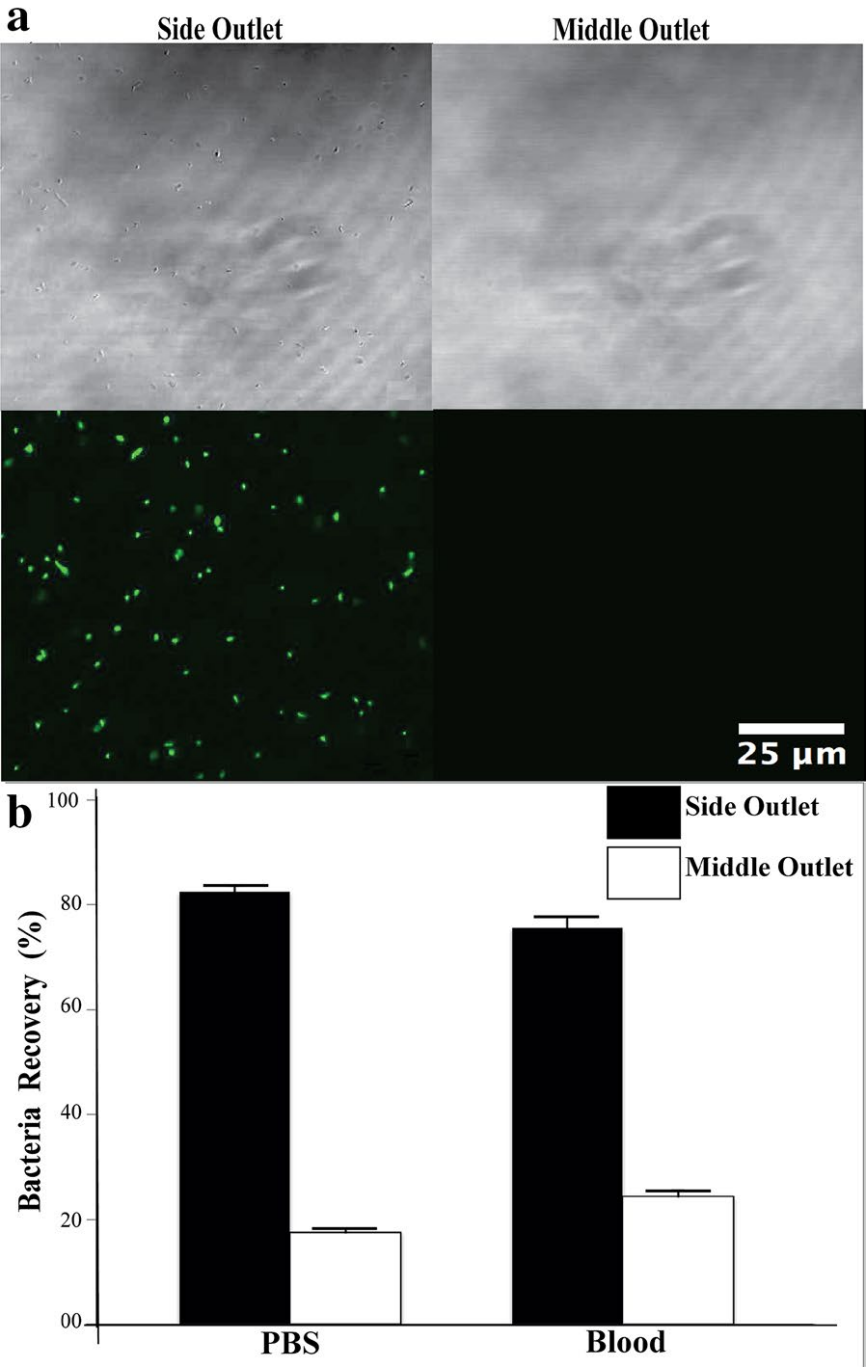
Bacteria separation from PBS and whole blood. **a** Separation of fluorescent tag expressed bacteria in PBS, bright field and fluorescent images from middle (M) and side (S) outlets. **b** Quantitative separation efficiency of bacterial spiked PBS and whole blood. The yield of bacteria collected at the side outlet was 82% for PBS and 76% for bacteria spiked in whole blood

Based on the flow conditions, we tested how *E. coli* bacteria will behave first using buffer and then spiked in whole blood. For the tests in PBS, we used fluorescent-tagged bacteria spiked at flow rates of 30 µl/h from side inlet and 500 ppm PEO was introduced at flow rate of 360 µl/h. As expected, the bacteria kept flowing along the channel wall and remain unaffected by normal stress due to its size (see Additional file 4: Figure S4). As can be seen in Fig. 5a, the sample collected from side outlet contains bacteria while there are no bacterial seen in sample collected from the middle outlet. For bacteria spiked in whole blood, the blood sample was introduced at 30 µl/h and the 500 ppm PEO at 360 µl/h. To quantify bacteria spiked in whole blood as well as in PBS, we used plating after collection of the fractions (Fig. 5b). For PBS, the 82% bacteria remained at the side outlet while for blood sample 76% of bacteria remained at the side outlet. The reduced bacteria recovery at the side outlets in whole blood sample compared to the PBS sample is mainly attributed to particle–particle interaction as well as the complex nature of the whole blood that could potentially make the bacteria stick to blood cells and migrate along to the middle outlet. We tested a range of different flow rates (covering those of Fig. 4c) and found the bacteria recovery from the side outlets to be relatively stable, about 81–82% for bacteria spiked in PBS and 75–76% for whole blood (see Additional file 5: Figure S5). Interestingly, the bacteria recovery remained high (73% at side outlet) when the blood flow rate was doubled from 30–60 µl/h.

Culture-independent, PCR based, detection of pathogens directly from the patient’s blood is attractive to accelerate the diagnostic process. However, the use of whole blood in assays designed to detect pathogen nucleic acid is challenging. An excess of human DNA may hamper the detection of pathogen genomic material or inhibit the PCR reaction [36, 37]. Furthermore, hemoglobin traces may also inhibit PCR-based amplification [38]. Therefore, molecular methods are often forced to use a relatively small volume of blood, which affects the sensitivity. As shown above (see Fig. 4c), our continuous flow sample preparation strategy significantly reduces the complexity by getting rid of majority RBCs and WBSs. However, for sepsis diagnostics, the amount of bacteria present in the blood is very low—in the order of 10–100 cfu/ml. Hence, to be clinically relevant it is imperative to further improve the method in order to recover all bacteria cells as well as improve the sample throughput.

In elasto-inertial microfluidics, the synergetic effect of visco-elastic forces and inertial forces are harnessed such that particles can migrate and occupy a single focusing point. This focusing phenomenon has been used for various applications, including sheathless cell ordering [39, 40] size based cell separation [33, 41, 42] and cell stretching measurements [43]. Nam et al. [33] used similar channel geometry as ours to separate platelets from diluted blood components with extremely high purity (close to 99.9%). However, the relatively slow flow rate combined with the use of diluted blood sample (less than 1% solid content) makes it un-applicable for bacteria separation applications. Here, we have demonstrated bacteria separation from undiluted whole blood. While the method is immediately applicable as sample preparation for MALDI-ToF MS based identification of microorganisms from positive blood cultures, for blood culture independent molecular diagnostics the method needs to process ml volumes blood sample. The relative low volumetric flow rate is an inherent limitation of elasto-inertial microfluidics since the synergetic effect of the elastic forces and inertial forces are ideal at moderate flow rates. For instance, even at volumetric flow rate of 60 µl/h tested in this work, it would take about 17 h to process 1 ml blood. One way to improve throughput is therefore through parallelization of the channels. Towards this, we have recently reported on a highly scalable, parallel-channel, microfabrication method for passive size-based particle separation [44]. Using the 16-channel parallel device [44], it would take only 1 h to process 1 ml. While outside the scope of this paper, we are currently working on combining the robust microfluidic fabrication process of parallel channels (64 channels), with bacteria separation based on elasto-inertial microfluidics. The method has potential value in clinical sample preparation applications for both molecular diagnostics as well as analysis by plating for antibiotic susceptibility.

## Conclusions

We demonstrated bacteria separation from whole blood based on elasto-inertial microfluidics. By harnessing the synergetic effect of elastic and inertial forces, we first demonstrate efficient particle separation where we could separate 5 µm particles from 2 µm at a yield of 95% for 5 µm and 93% for 2 µm particles at the respective outlet fractions. Furthermore, we successfully demonstrated bacteria isolation from undiluted whole blood by selectively migrating the larger blood cell components from the sidewalls towards the centerline for separation. 76% of the bacteria were recovered at the side outlet while 92% of the WBCs could be separated into the middle outlet. The passive, label-free bacteria separation method is very promising and has great potential as stand-alone sample preparation method or integrated into lab-on-chip system for molecular and phenotypical based sepsis diagnostics.

## Methods

### Microfluidic device fabrication

The polydimethylsiloxane (PDMS) microfluidic chips with two inlets and two outlets were fabricated on a master mould that was produced through photolithography on a silicon wafer using SU-8 negative resist. PDMS Sylgard 184 was then poured onto the SU-8 master in a 10:1 ratio, degassed, and cured at 65 °C overnight. The PDMS slab was cut, the holes for inlets and outlet were punched, and covalently bonded to glass slides using oxygen plasma (CUTE Femto Science Co. Korea) treatment. The following cross section dimension (width × height) were fabricated: 50 µm × 65 µm; 100 µm × 65 µm and 150 µm × 65 µm.

### Experimental

Suspension of different fluorescent polystyrene particles (2, 5 and 10 µm) were prepared in Phosphate Buffered Saline (1× PBS). Poly (ethylene oxide) (Sigma Aldrich, St Louis), M_w_ = 2,000,000 was prepared at different concentrations of 250, 500, 750 and 1000 ppm in 1× PBS. To mimic the sepsis blood samples, blood samples obtained from healthy blood donors were spiked with bacteria (~10^6^ cfu/ml). As a model strain, gram-negative *E. coli* (strains BL21-A1) cultured in liquid medium, collected at the mid-log phase, washed with PBS, was used to spike the blood samples.

For the particle based experiments, the solution containing the particles were introduced into side inlet using a syringe pump (Harvard apparatus PHD 2000, Harvard Apparatus, USA) and Non-Newtonian fluid of PEO was introduced into middle inlet by syringe pump (NEMESYS, Cetoni Gmbh, Germany). The particles collected from the side and center outlet were analyzed using Coulter counter for quantification. For the bacteria related work, initially PBS (1×) solution was spiked with bacteria. This was followed by whole blood experiments spiked with bacteria. The experimental procedure was similar as for the particle suspension. Coulter counter was used to quantify the white blood cells (WBCs) while plating was used to quantify the bacteria.

## Additional files

**Additional file 1: Figure S1.** Theoretical background of elasto-inertial microfluidics. In non-Newtonian viscoelastic fluids, elastic forces (F_e_) acts on suspended particles directing them towards the centre and four corners of microchannel. This migration of particle is the result of non-uniform normal stress differences. For the shearing flow regime these stress are defined as first (N_1_ = τ_xx_ − τ_yy_) and second normal stress (N_2_ = τ_yy_ − τ_zz_) differences. Here τ is the stress tensor and subscript x, y and z are denoting direction of flow, direction of velocity gradient and z is the direction of vorticity respectively. Since for most of the polymeric solution the N_1_/N_2_ ratio is less than 0.1, the effect of N_2_ could be neglected while the elastic force, F_e_, on the particle is scaled with effect of N_1_ which varies depending upon the size of particle (F_e_ ~ a^3^ ∇N_1_). This elastic effect could be defined by an important dimensionless parameter known as Weissenberg number (W*i*); which is the ratio of viscous forces to elastic forces the fluid experiences. W*i*, with some simplifications, is defined as the product of shear rate (ϒ) to the relaxation time (λ) of non-Newtonian fluid (λ): W*i* = λ ϒ = λ2U/w = 2Q/hw^2^. Q is the total flow rate and “w” and “h” are the width and height of microchannel respectively. In flow through rectangular channels, one-way to reduce the particle focusing points (away from the four corners of microchannel) towards only the centre channel is by increasing the flow rate such that inertial forces start to act on particles—and hence the term “elasto-inertial microfluidics”. In inertial microfluidics, shear-induced lift force (F_L,S_) due to shear gradient of the parabolic flow profile acts on suspended particles towards the wall, while wall induces a lift force that directs the particles away from the wall i.e. towards the centre of flow stream (F_L,w_). Due to the balance of these lift forces the particle attains a certain equilibrium position within rectangular channel. The net lift force (F_L_) on particle is derived by combination of both shear-induced lift force, F_L,S_ and F_L,W_: F_L_ = f_c_ ρU^2^ a^4^/D_h_^2^ = f_c_ Rp^2^ µ^2^/ρ. Here, ρ is the density, U is the mean velocity and µ is dynamic viscosity of the fluid. Particle diameter is denoted by a, and D_h_ [=2wh/(w + h)] is the hydraulic diameter of microchannel. The coefficient of inertial lift force, f_c_, is dependent on position of particle in the microchannel and Reynolds number (Re = ρUD_h_/µ), which defines the scale of these net forces within channel. Particle’s Reynolds number Rp is another important dimensionless number [Rp = Re (a/D_h_)^2^]. Elasto-inertial focusing is achieved by optimizing the flow rate of the non-Newtonian fluid, such that both inertial (F_L_) and elastic (F_e_) forces act to focus relatively larger particle into a single stream in 3D in flow through rectangular channels.

**Additional file 2: Figure S2.** Particle defocussing due to shear thinning effect as flow rate is increased. 5 µm particle suspended in PBS (1×) is introduced in micro channel with 500 ppm PEO non-Newtonian at (a) total flow rate = 11 µl/min where 5 µm particle flow rate = 1 µl/min; Rp = 0.0132 (b) total flow rate = 12 µl/min, 5 µm particle flow rate = 2 µl/min; Rp = 0.0144, and (c) total flow = 13 µl/min, 5 µm particle flow rate = 3 µl/min; Rp = 0.016, It could be seen that at (c) Rp 0.016 focused streams broadened indicating the shear thinning effect and dilution of non-Newtonian fluid.

**Additional file 3: Figure S3.** 5 µm particles focusing behavior mapped for different concentrations of PEO (non-Newtonian fluid concentration of 250, 500, 750 and 1000 ppm), unfocussed (red), partially focused (yellow) and focused (green) particle positions with respect to center of micro channel are shown.

**Additional file 4: Figure S4.** Confocal image of bacteria flowing inside microchannel. The fluorescent tagged spiked bacteria in PBS was introduced into PEO based elasto-inertial flow, and no displacement of bacteria is observed close to the outlet in elasto inertial flow, presumably due to the insignificant magnitude of forces on bacteria.

**Additional file 5: Figure S5.** Elasto-inertial microfluidics based bacteria separation from PBS (a) and whole blood sample (b) for a range of flow rates. The bacteria recovery from the side outlet is relatively constant (81–81% for PBS and 75–76% for whole blood) over the different flow rates. When the flow rate of the blood sample is doubled (from 30 to 60 µl/h), the recovery rate of bacteria remained relatively high (73%).
