# Supplementary figures and images for: Elasto-inertial microfluidics for bacteria separation from whole blood for sepsis diagnostics
Source: J Nanobiotechnology. 2017 Jan 4;15:3. doi: 10.1186/s12951-016-0235-4 (PMC5210221; doi:10.1186/s12951-016-0235-4)

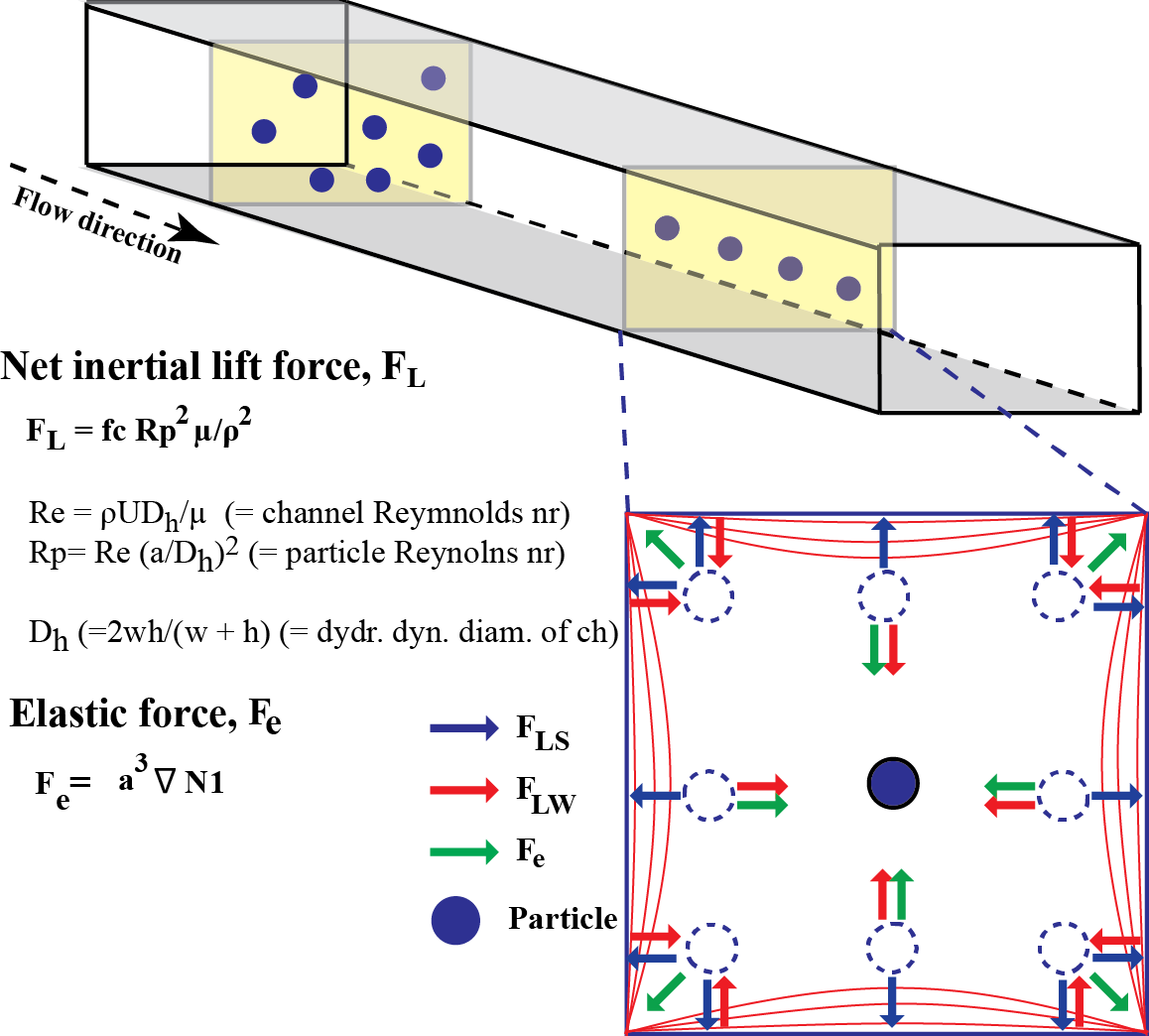

Supplement: Supplementary file 1 — Additional file 1: Figure S1. Theoretical background of elasto-inertial microfluidics. In non-Newtonian viscoelastic fluids, elastic forces (Fe) acts on suspended particles directing them towards the centre and four corners of microchannel. This migration of particle is the result of non-uniform normal stress differences. For the shearing flow regime these stress are defined as first (N1 = τxx − τyy) and second normal stress (N2 = τyy − τzz) differences. Here τ is the stress tensor and subscript x, y and z are denoting direction of flow, direction of velocity gradient and z is the direction of vorticity respectively. Since for most of the polymeric solution the N1/N2 ratio is less than 0.1, the effect of N2 could be neglected while the elastic force, Fe, on the particle is scaled with effect of N1 which varies depending upon the size of particle (Fe ~ a3 ∇N1). This elastic effect could be defined by an important dimensionless parameter known as Weissenberg number (Wi); which is the ratio of viscous forces to elastic forces the fluid experiences. Wi, with some simplifications, is defined as the product of shear rate (ϒ) to the relaxation time (λ) of non-Newtonian fluid (λ): Wi = λ ϒ = λ2U/w = 2Q/hw2. Q is the total flow rate and “w” and “h” are the width and height of microchannel respectively. In flow through rectangular channels, one-way to reduce the particle focusing points (away from the four corners of microchannel) towards only the centre channel is by increasing the flow rate such that inertial forces start to act on particles—and hence the term “elasto-inertial microfluidics”. In inertial microfluidics, shear-induced lift force (FL,S) due to shear gradient of the parabolic flow profile acts on suspended particles towards the wall, while wall induces a lift force that directs the particles away from the wall i.e. towards the centre of flow stream (FL,w). Due to the balance of these lift forces the particle attains a certain equilibrium position within rectang [file 12951_2016_235_MOESM1_ESM.png]

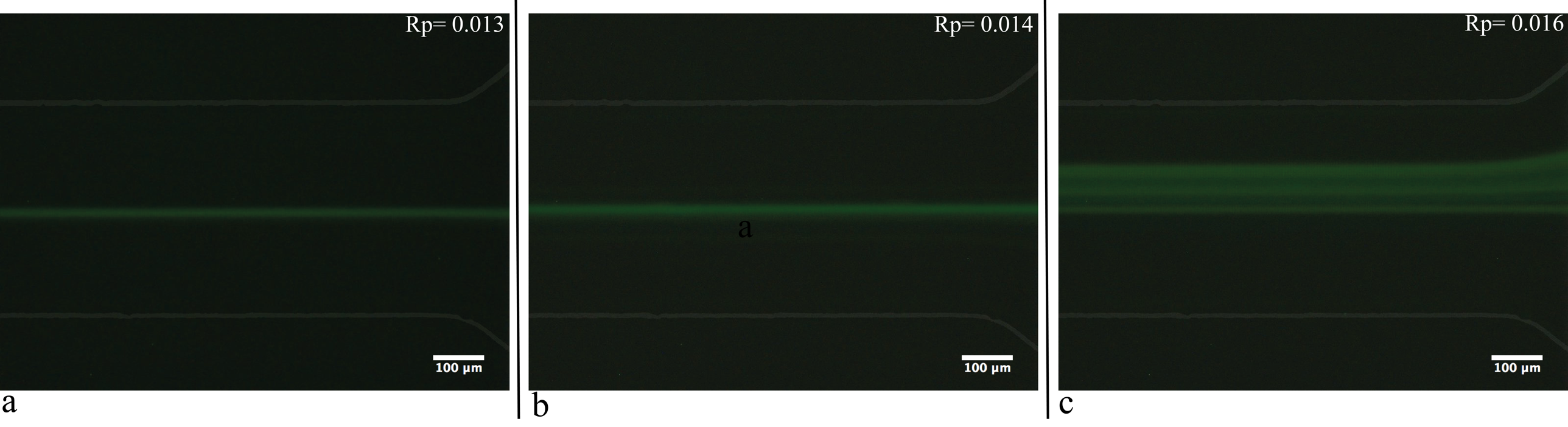

Supplement: Supplementary file 2 — Additional file 2: Figure S2. Particle defocussing due to shear thinning effect as flow rate is increased. 5 µm particle suspended in PBS (1×) is introduced in micro channel with 500 ppm PEO non-Newtonian at (a) total flow rate = 11 µl/min where 5 µm particle flow rate = 1 µl/min; Rp = 0.0132 (b) total flow rate = 12 µl/min, 5 µm particle flow rate = 2 µl/min; Rp = 0.0144, and (c) total flow = 13 µl/min, 5 µm particle flow rate = 3 µl/min; Rp = 0.016, It could be seen that at (c) Rp 0.016 focused streams broadened indicating the shear thinning effect and dilution of non-Newtonian fluid. [file 12951_2016_235_MOESM2_ESM.png]

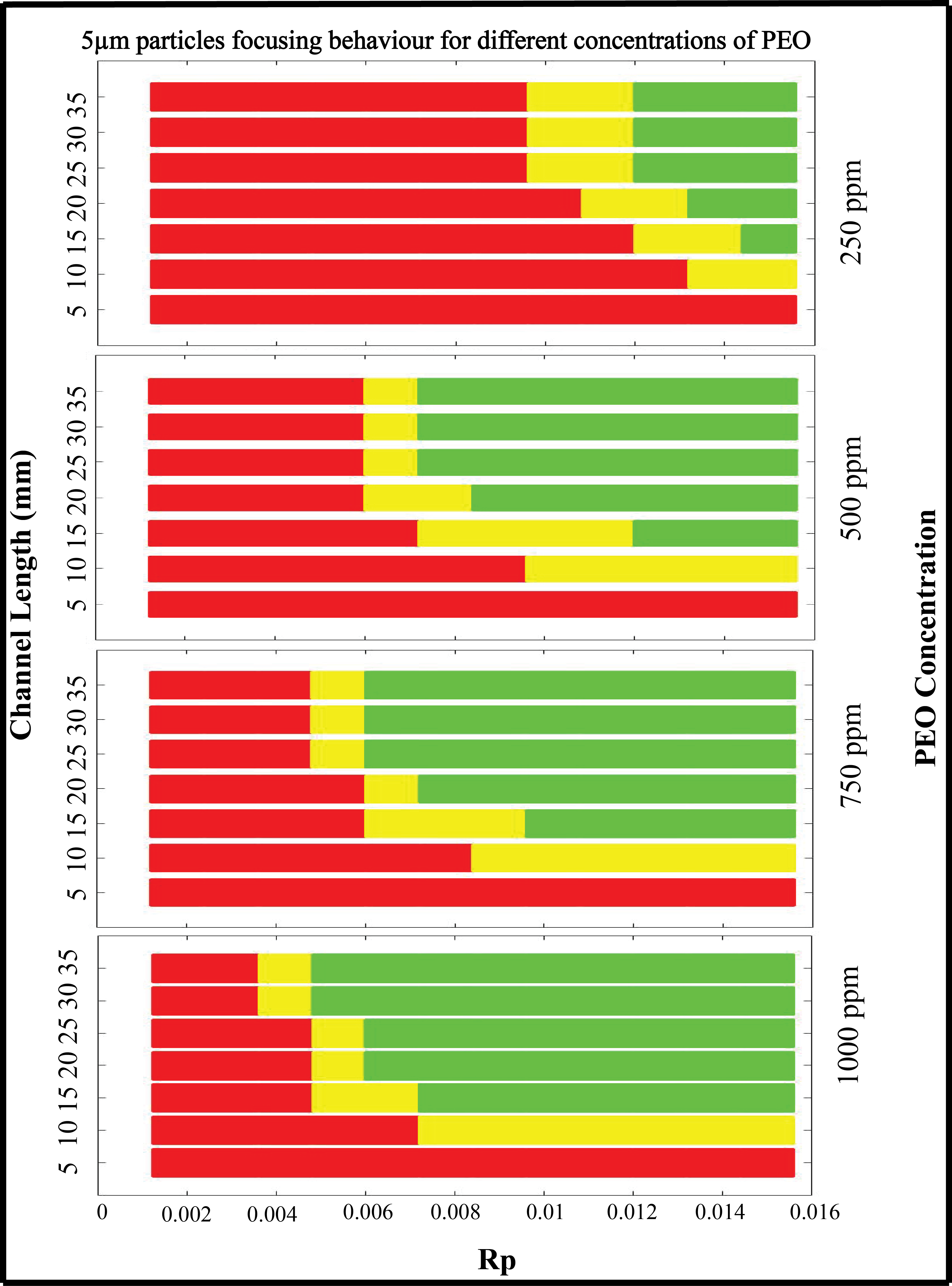

Supplement: Supplementary file 3 — Additional file 3: Figure S3. 5 µm particles focusing behavior mapped for different concentrations of PEO (non-Newtonian fluid concentration of 250, 500, 750 and 1000 ppm), unfocussed (red), partially focused (yellow) and focused (green) particle positions with respect to center of micro channel are shown. [file 12951_2016_235_MOESM3_ESM.png]

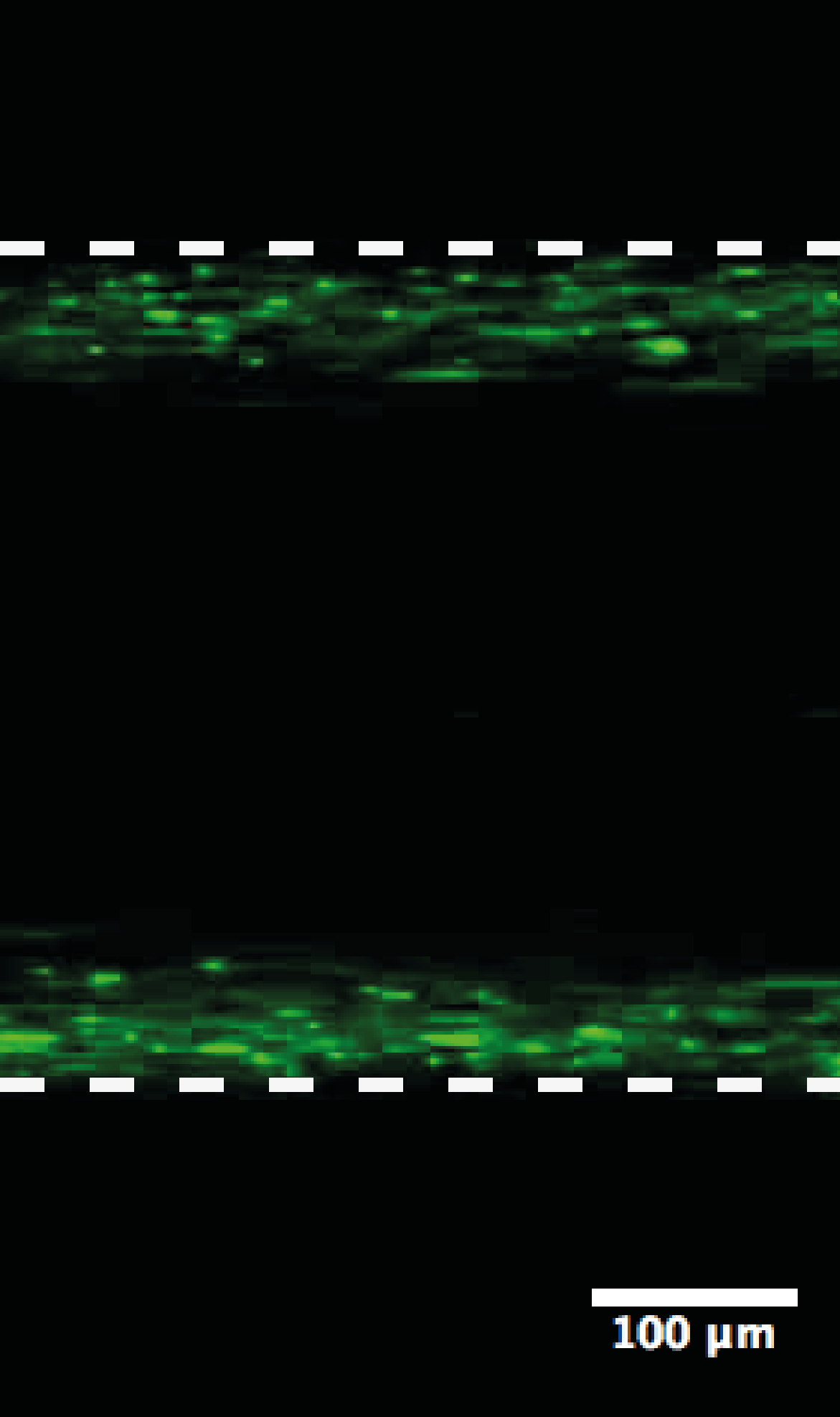

Supplement: Supplementary file 4 — Additional file 4: Figure S4. Confocal image of bacteria flowing inside microchannel. The fluorescent tagged spiked bacteria in PBS was introduced into PEO based elasto-inertial flow, and no displacement of bacteria is observed close to the outlet in elasto inertial flow, presumably due to the insignificant magnitude of forces on bacteria. [file 12951_2016_235_MOESM4_ESM.png]

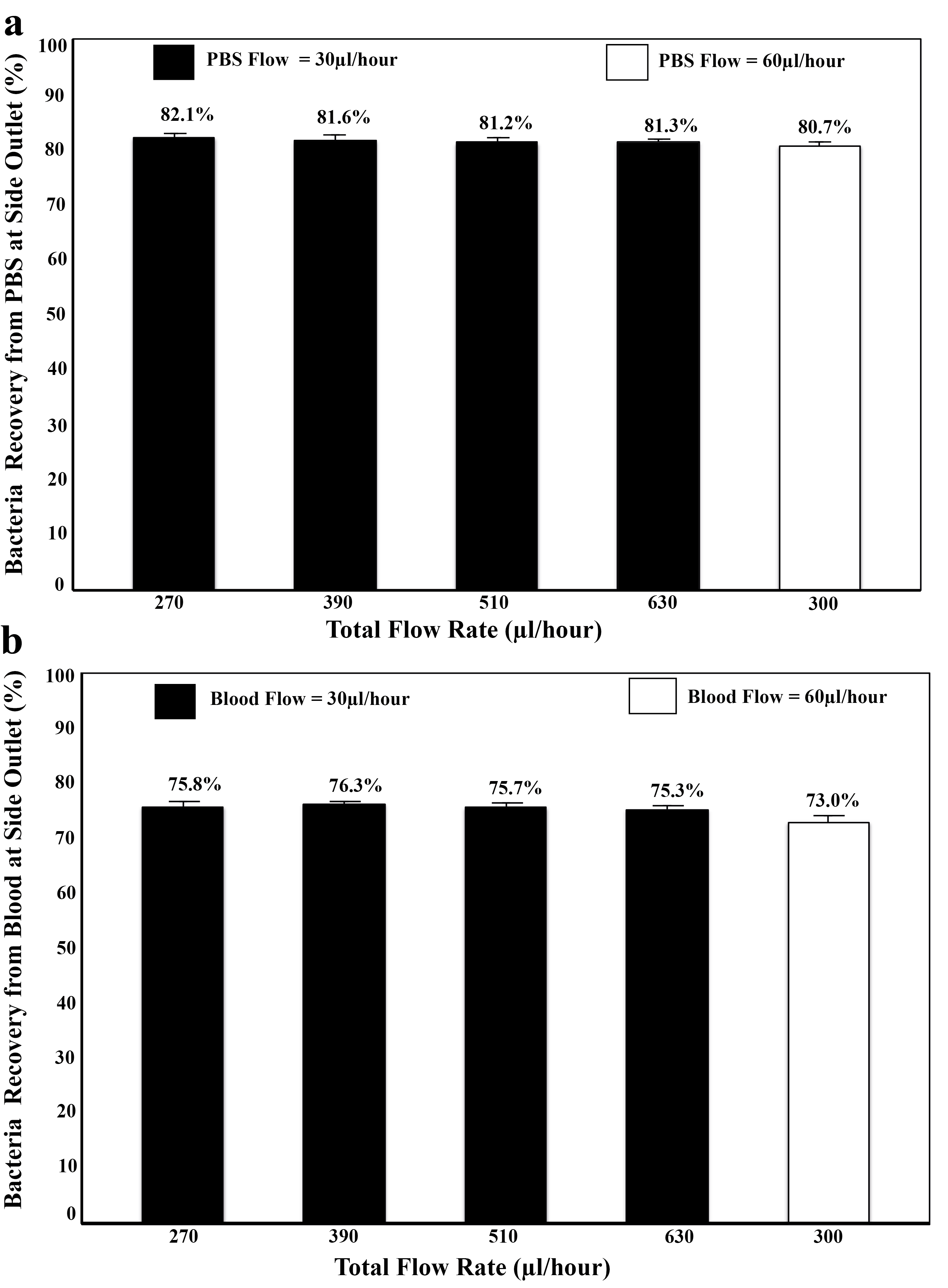

Supplement: Supplementary file 5 — Additional file 5: Figure S5. Elasto-inertial microfluidics based bacteria separation from PBS (a) and whole blood sample (b) for a range of flow rates. The bacteria recovery from the side outlet is relatively constant (81–81% for PBS and 75–76% for whole blood) over the different flow rates. When the flow rate of the blood sample is doubled (from 30 to 60 µl/h), the recovery rate of bacteria remained relatively high (73%). [file 12951_2016_235_MOESM5_ESM.png]
